# Tidal range electricity generation: A comparison between estuarine barrages and coastal lagoons

**DOI:** 10.1016/j.heliyon.2022.e11381

**Published:** 2022-11-04

**Authors:** David Vandercruyssen, Simon Baker, David Howard, George Aggidis

**Affiliations:** a Lancaster University Renewable Energy Group and Fluid Machinery Group, Engineering Department, Bailrigg, Lancs, Lancaster, LA1 4YR, UK; b UK Centre for Ecology & Hydrology, Lancaster Environment Centre, Library Avenue, Bailrigg, Lancaster, LA1 4AP, UK

**Keywords:** Renewable energy, Tidal range, Tidal barrage, Morecambe bay barrage, North Wales lagoon

## Abstract

The potential power from coastal tidal range is becoming better appreciated due to the need to mitigate global warming. Great Britain (GB) is ideally situated to exploit tidal power but currently has no operational systems. Historically, estuaries have been proposed as sites for barrages, but more recently coastal lagoons are favoured due to a lower environment impact. To contrast the differences between barrages and lagoons two potential schemes are analysed using the Lancaster 0-D Tidal Range Model. Both schemes were analysed with a range of turbine numbers and generator ratings. The schemes are compared in terms of energy generation, flood protection, navigation, and selected environmental impacts.

The analysis indicates that the schemes are not categorically different, characterised by the shape and alignment of the impoundment. Barrages impoundments across estuaries are generally shorter than lagoons impounding similar volumes, with lower civil engineering costs. Whilst estuaries tend to have slightly higher tidal ranges, they also create unique ecological conditions with diverse natural ecosystems that are increasingly valued. The analysis shows that 2-way generation and pumping can match the full tidal range and help preserve inter-tidal areas.

## Highlights

• Estuary barrage and coastal lagoon energy generation and environmental impact.
• Two-way generation & pumping limits prevents loss of intertidal area.
• Flood protection from storm surges and sea-level rise.
• Annual electricity generation for various generator ratings and sluice ratios.
• Comparison of two case study sites with identical intertidal range.

## 1 Introduction

Interest in tidal range energy generation has grown recently due to the need for more renewable energy. Electricity demand in the United Kingdom (UK) is forecast to double as electricity replaces petrol and diesel to power transport [1]. It may quadruple if electricity replaces natural gas for domestic heating or is used to generate hydrogen for transport and heating. There are now several proposed tidal range schemes around the coastline of Great Britain (GB) [2, 3].

The timing and amplitude of tidal range power generation is predictable many years ahead as it is based on the cycles of the moon and the earth's orbit around our sun. Tidal range schemes can convert energy over 4 separate periods each lunar day when generating electricity on both the incoming (flood) and outgoing (ebb) tides. The periodic and predictable nature of tidal energy conversion is termed cyclically intermittent. The generating period varies at a first approximation with the reservoir area, the height of the tide and the discharge capacity of the turbines and sluices. For a given barrage or lagoon, the duration varies significantly between neap and spring tides in the order of 2–4 h. Spring tides also generate significantly more power due to their higher head. High tides occur at different times of day in different locations around GB; a chain of carefully selected sites could produce some measure of continuous daily generation [4]. For today's electricity demand profile, and, without efficient storage, conventional wisdom prescribes that generation should match demand. However, the use of electric vehicles is predicted to preferentially increase overnight demand [5] and act as a mass storage system. The use of large batteries and hydrogen production could be linked directly to the tidal generation programme to balance supply and demand.

In this paper, the term tidal barrage represents a barrier across the estuary of a major river with capacity to allow the tides to move in and out. Typically, these schemes encompass a large tidal volume for a relatively short barrage. The term coastal lagoon represents a lagoon formed by a barrier impounding a volume of sea adjacent to a length of coastline. The length of bund relative to enclosed area is usually lower for an estuarine barrier than a coastal lagoon. There are several such schemes proposed for the west coast of GB, some also include flood protection, but currently no scheme that has progressed beyond the outline stage.

The popular perception is that estuarine barrages and coastal lagoons are different in construction, operation and performance and the schemes are often viewed as competitive alternatives, especially by developers, administrators and government (e.g. Hendry review [2]). The aim of this paper is to address the preconceptions and contrast the wider benefits of each style of scheme. Construction costs will be discussed in subsequent papers by the authors.

Estuarine barrages have to date been opposed by environmental lobby groups. British estuaries have the greatest levels of designation and protection in the country, making development seem virtually impossible. Recently there has been greater interest in tidal lagoons as they are considered to have a lower environmental value and show greater public approval. This paper shows that much of the environmental opposition is outdated and that tidal range can help preserve important habitats.

The Hendry Review [2] found that there is clear evidence that large scale tidal lagoons can play a significant role in providing sustainable power to the British economy. Whilst it supports the idea of a pathfinder project it argues that it should be seen as a power only scheme and judged on its financial costs and returns when generating electricity. The Review also called for positive Government action setting up a competitive tender process for large scale schemes. The involvement of public funds will require transparency and fairness in the assessment of different schemes. The simulations described below question the preconceptions and demonstrate how the power output for different sites can be compared.

Morecambe Bay was chosen to demonstrate an estuarine barrage, following the work of Baker [6], and North Wales was candidate for a lagoon, following the work of Xue [7]. To allow the schemes to be legitimately compared they were reanalysed using an identical 0-D modelling method [6, 8] and with matching pumping objectives, i.e. to maintain the existing tidal ranges within the impoundments to minimise disruption to the intertidal zones. To remove the effects of the tide differences, modelling for each site is performed using both its own site-specific tidal data and the tidal regime for the other site. Each scheme was analysed with various numbers of turbines and generator rating.

**Figure 1:**
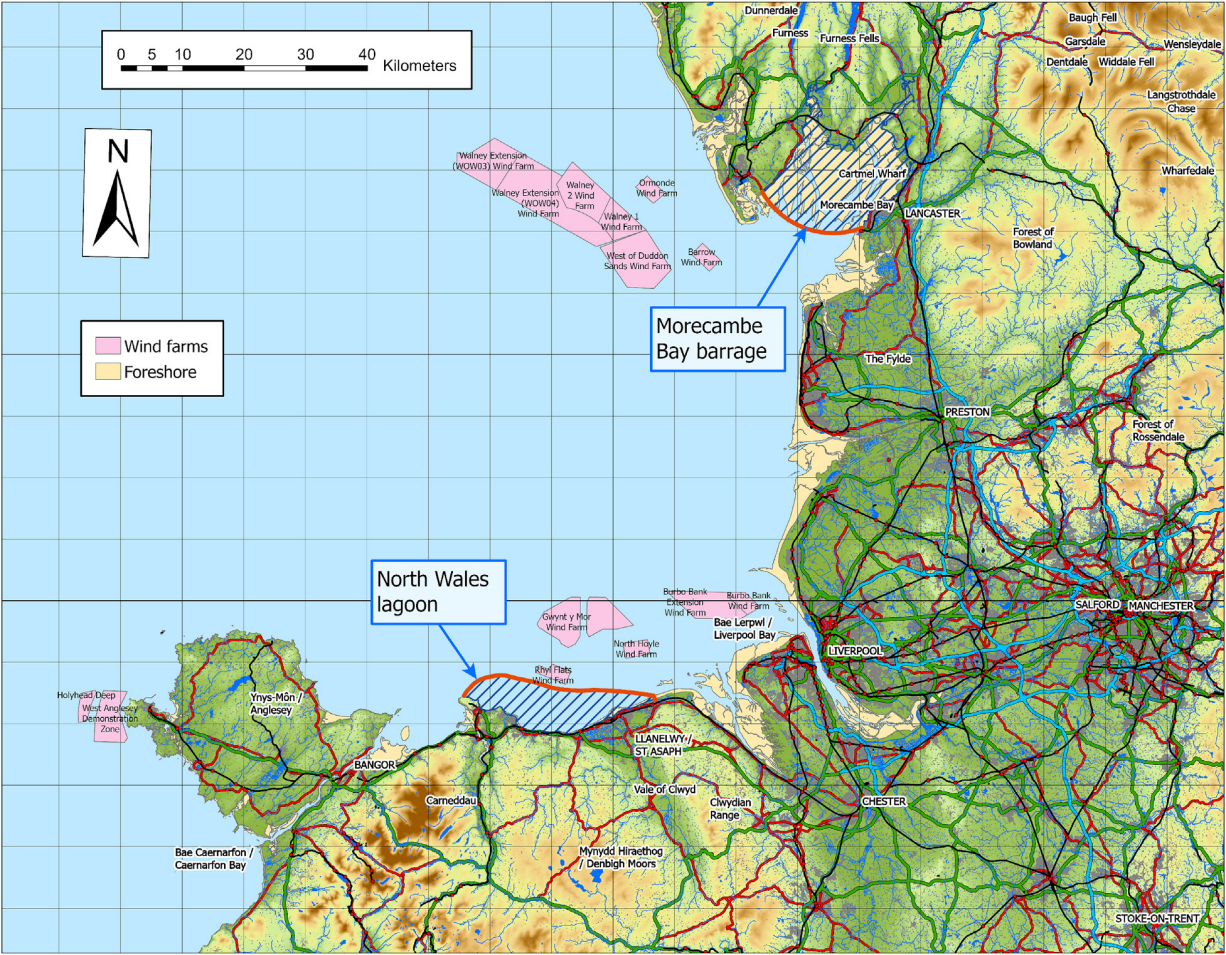
Location map showing the position of proposed Morecambe Bay barrage and North Wales tidal lagoon.

Figure 1 shows the relative locations of the two demonstrator schemes. They were selected as they both have seen commercial interest in development and are geographically neighbours on the Irish Sea. Morecambe Bay has higher tidal range than North Wales. The curved red lines represent possible locations for the bunds and the hashed blue lines the impounded areas at high tide.

### 1.1 Flood protection

If barrages were solely designed for electricity generation the barrage height would only need to be about as high as the highest tide with an allowance for storm surges. Occasional overtopping would not be harmful and can be designed for. If the barrage includes a public road, or is intended to offer flood protection, the crest level will need to be higher to allow for 150-year storm surges, waves, and predicted sea level rise. For roads and flood protection the barrage height will need to be the sum of the highest tide, maximum storm surge (2 m typically), freeboard for waves (1m typically) and sea level rise. The Institution of Mechanical Engineers 2019 report [9] recommended “prepare for a minimum of a 1 m rise in sea level this century but plan for 3 m of rise”. The bund design will need to include provision for raising the height at a future date as the extent of sea level rise becomes clear.

Coastal and estuarine flooding tends to occur when high spring tides, low air pressures and strong winds coincide. This situation exacerbates terrestrial riverine flooding as rivers ‘back up’ against incoming tides. When such conditions are forecast the turbine and sluice gates can be closed to maintain the impounded water level below river levels. This will create space for rivers to drain into the impoundment in the normal way. In extreme cases the turbines can pump water out of the impoundment. The impounded water will not exceed normal high tide level. When the sea level reduces sufficiently normal generation can resume. The barrage will also “break” the waves and greatly reduce the height of those impacting the shore. Thus, the coastal areas and inland riverbanks will be protected from the sea.

### 1.2 Transport

Every scheme will have its own arrangements for transport and navigation but in general an estuarine barrage can provide a road link between the two shores. The proposed 17-km road link across Morecambe Bay has been described as providing “An estimated nine million annual crossings – reducing travel distance by 50% and journey time by 75%, with fuel savings of 750,000 L annually” [10]. Conversely large estuaries are often used by shipping and the locks will increase transit times.

## 2 Demonstration schemes

Morecambe Bay, an example of an estuarine barrage; this scheme is promoted by Northern Tidal Power Gateways (NTPG) [6, 11]. The coastal lagoon case used for comparison is along part of the North Wales coast and is being promoted by North Wales Tidal Energy (NWTE) [12]. Each scheme is reported to have a similar capital cost of around £7 billion.

### 2.1 Morecambe Bay, an estuarine barrage

Morecambe Bay is situated on the coast of northwest England between the counties of Cumbria to the north and Lancashire to the south. Barrow-in-Furness is at the north-western end of the proposed barrage; Heysham, Morecambe and Lancaster at the south-eastern end. Most of the land between Barrow and Carnforth is low hills drained by the Leven and Kent rivers; their fluvial valleys originate in the southern Lake District fells and the Pennines.

**Figure 2:**
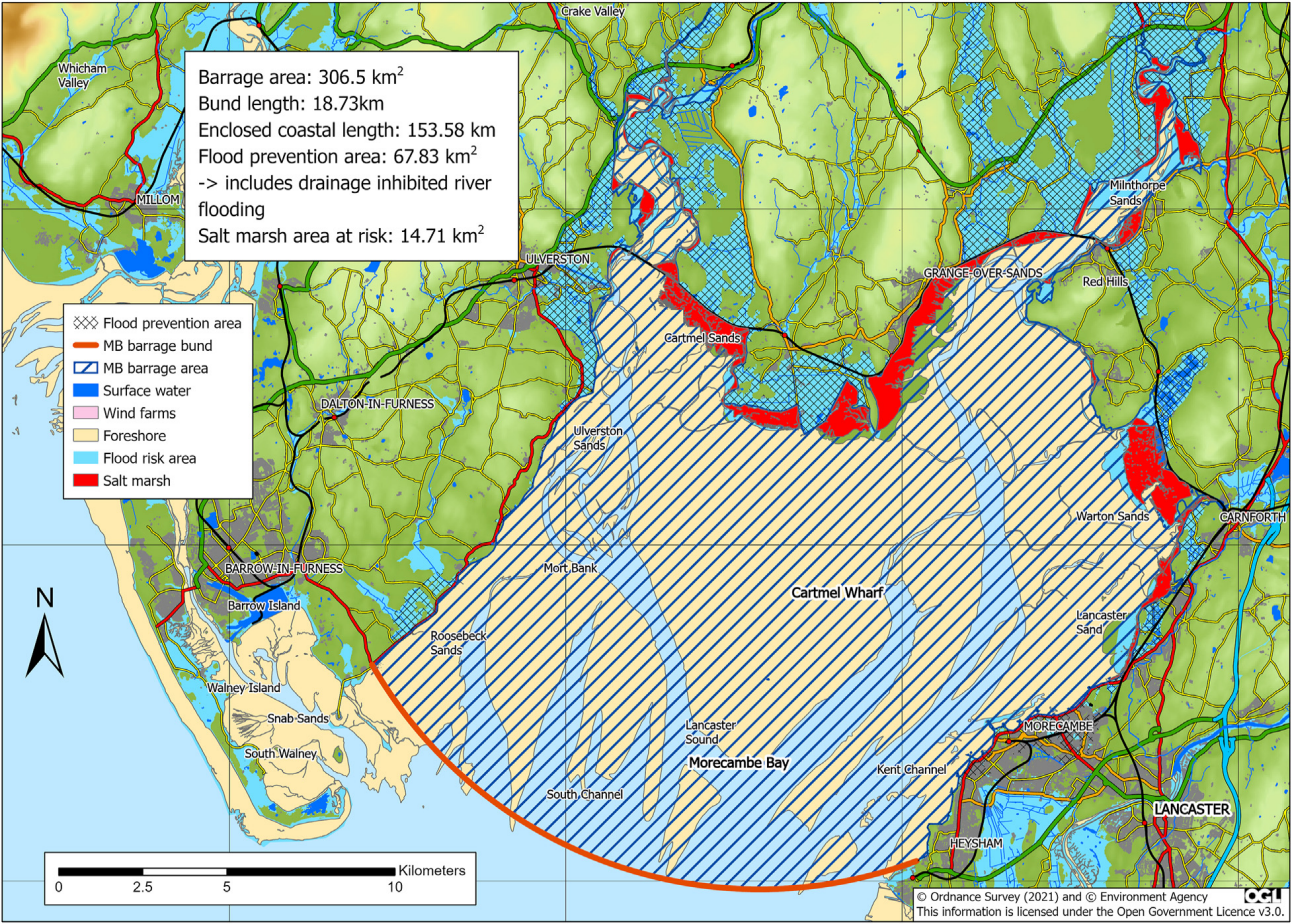
Proposed Morecambe Bay barrage area indicating existing terrestrial flood threat, and the intertidal impoundment.

Figure 2, after Baker [6], shows the impoundment area. The generating capacity is a fundamental parameter affecting the operation and performance of the barrage and is determined principally by the number and size of the turbines. For the Baker study, the turbine runner diameter was set at 8m and the size of sluice gates was set at 15 × 15m.

#### 2.1.1 Flood risk around Morecambe Bay

The dark blue crosshatched areas in Figure 2 represent the areas currently at risk from a 1:150-year flood. A barrage across Morecambe Bay will offer protection from tidal flooding to Ulverston, Grange-over-sands, Milnthorpe, Carnforth, Morecambe, parts of Lancaster and several villages. Considerable sums of money have already been spent on marine flood defence in the way of levees, causeways and pumping stations. To prevent sea level rise disrupting transport or causing damage to property and infrastructure many will require strengthening or replacing. Riverine floods within the catchment (e.g. the River Kent in 2016) are occurring more regularly and have caused catastrophic damage [13]. A barrage can be closed before high tide to allow rivers to drain.

In terms of geometry, estuaries usually will have longer shoreline borders than a coastal lagoon (approximately two thirds of the impounded water body's boundary as opposed to less than half of a lagoon). Consequently, more human stock and infrastructure is expected to be protected from the risk of flooding in an estuarine location; this is compounded since estuaries were commonly selected sites for settlement due to the presence of the waterway that may need crossing points and offers transport (river and sea).

### 2.2 North Wales, a coastal lagoon

**Figure 3:**
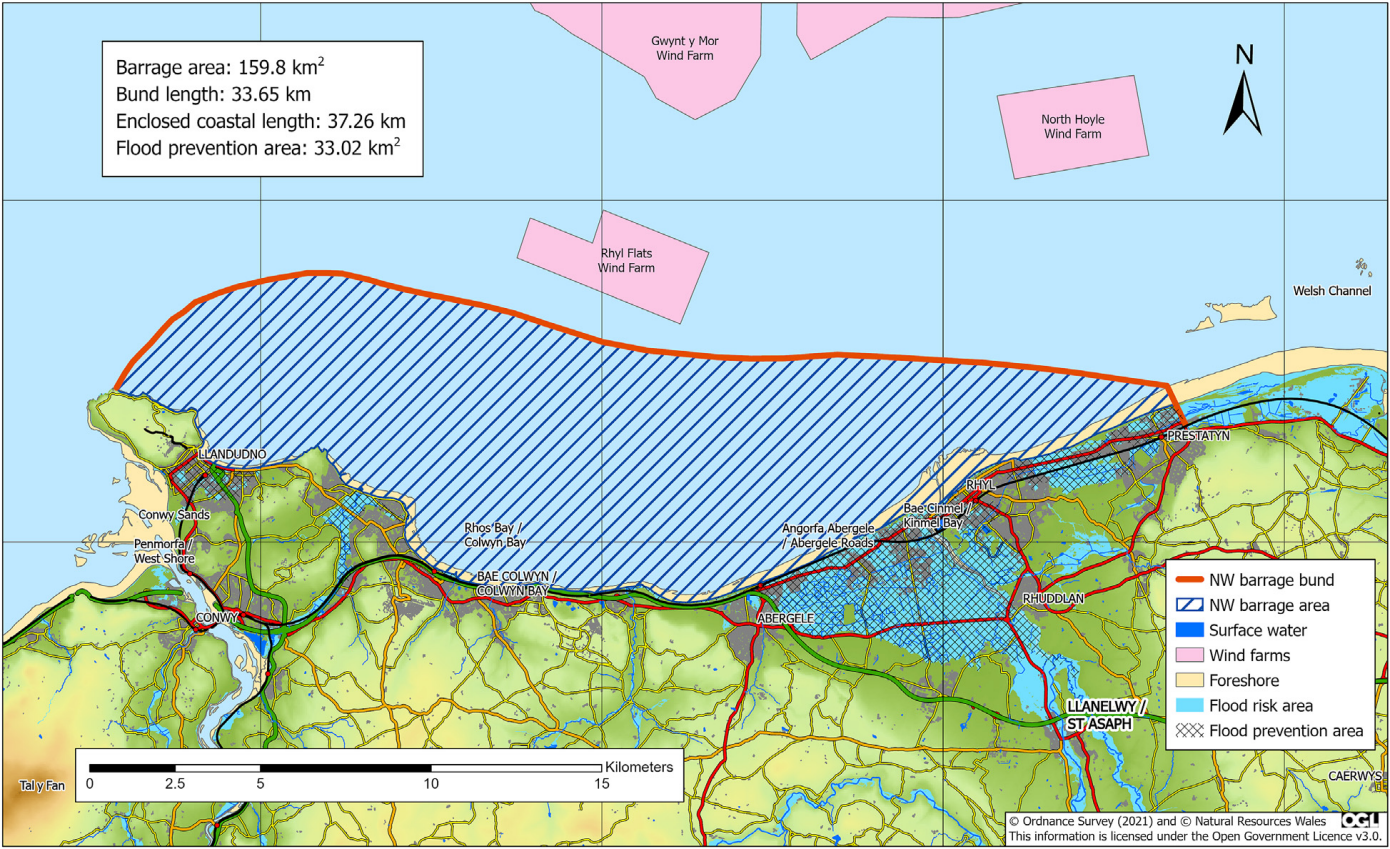
Assumed location of North Wales Coastal Lagoon showing impoundment and areas at risk from 1 in 150-year flood.

The location and bathymetry are those used by Xue [7] (Figure 3), with an impoundment area of 160 km^2^ and barrage length of 34 km. Xue carried out both 0-D and 2-D analysis assuming 20-MW turbines with 8m diameter runners.

The nearest national tidal monitoring station is at Llandudno, which will be enclosed at the western end of the impoundment. Other partial tide recording stations are at Colwyn Bay (high tide only) and the Port of Mostyn. The partial information from Colwyn Bay and the Port of Mostyn show the tidal range increases slightly towards the east and high tide is slightly later than Llandudno times. Specific tide and wave monitoring buoys will need to be installed as part of the feasibility study. For this analysis, the authors take the chart tide levels from Llandudno.

NWTE are proposing an installed capacity of 2.0–2.5 GW using 20-MW bulb turbines. The exact number of turbines will depend on the balance between cost and generation output. The turbines may be grouped into several turbine houses along the western half of the barrage where the water depth is greater. The final locations can be positioned to give the best environmental outcomes by 3-D modelling when specific site data is available.

#### 2.2.1 Flood risk for North Wales

There are several areas of low-lying land that will be protected by the barrage. The light blue areas in Figure 3 show the extent of flooding from a 150-year event. It will be necessary to “close the gap” by joining the east end of the barrage to the A548 just east of Prestatyn, where the ground level is above 6.0 m OD. The area at risk from flooding includes several coastal towns including St Asaph which have a history of flooding; also, the main London to Holyhead railway line is threatened.

The mean high-water spring (MHWS) at Llandudno is 3.51 m Ordnance Datum (OD) Newlyn. Added to this there could be storm surges of up to 2 m, as shown for Morecambe Bay by Baker [6]. Waves will also add to maximum water levels. If average sea level rise much above 2m the flood protection will be limited without major reinforcement inland or the construction of a barrage across the river Dee.

## 3 0-D modelling, reservoir model and tidal information

The 0-D model is relatively simple and is based on the conversion of potential energy to kinetic energy. Potential energy is a function of the difference in surface level between the two bodies of water, this is termed hydraulic head or simply its head. Kinetic energy is a function of the velocity of water flowing through the turbine when generating. The equations describing power generation are those published by Aggidis et al. [8, 14]. The efficiency of the turbine is unique to each design and is usually described as a graph with axis of unit water discharge and rotational speed. Lines joining equal efficiency appear as contours, giving the name of hill chart. The hill charts are usually confidential, but the previous references include the hill chart for the Andritz 3-blade low head bulb turbine, similar to that used at Sihwa [15]. The turbine model is based on the Andritz turbine hill chart [8, 14, 16, 17] with triple regulation and various pumping scenarios, as described by Baker [6]. The implementation also employs an optimisation scheme to maximise annual energy production (AEP) by adjusting the operational parameters (starting heads, turbine speed, etc.) whilst satisfying pumping limits where possible. Any change to the number and size of turbines, power rating, sluice capacity, tide, sea-level, pumping limits, reservoir etc. requires the operation to be re-optimised. 0-D modelling ignores any hydrodynamic effects and assumes changes in the reservoir volume are distributed instantly across the impound surface area, i.e., the time for water to flow across the impoundment and the effects this might have on the head at the turbines is ignored.

Two site specific inputs are required to the model: the tide and reservoir definition. The method used here divides the input tidal sequence into short time steps and for each step determines the levels either side of the barrage, the operating mode (ebb or flood hold, sluice, pumping, or generation), and calculates the power and flow rate. Thus, the tide sequence is required as input, either as a furrier series of time and level, or read directly from tide tables [18, 19]. The latter reference can export tide levels at 10-minute intervals to a spreadsheet which can be read by the model.

Tide data from measuring stations at Llandudno and Heysham are used for the modelling at North Wales and Morecambe Bay respectively, see Table 1. Of note is the lower tidal range at North Wales (15% lower) than at Morecambe Bay, which limits the available generating head and in turn limits the power.

**Table 1:** Closest tidal gauging stations to the proposed North Wales tidal lagoon and Morecambe Bay barrage. [18].

			Llandudno	Heysham
		Latitude	53°19.908′N	54°1.908′N
		Longitude	3°49.494′W	2°55.224′W
		Grid ref	SH 7855 8319	SD 3982 5993
		Earliest data	1994	1964
Tide Levels (m, chart)	HAT	Highest astronomical tide	8.59	10.76
	LAT	Lowest astronomical tide	−0.42	0.22
	MHWS	Mean high water springs	7.36	9.67
	MHWN	Mean high water neaps	5.97	7.49
	MLWN	Mean low water neaps	2.20	3.05
	MLWS	Mean low water springs	0.48	1.18
	MSR	Mean spring range	7.20	8.49
	MNR	Mean neap range	3.77	4.44
		Chart Datum relative to OD Newlyn	−3.85	−4.90

**Figure 4:**
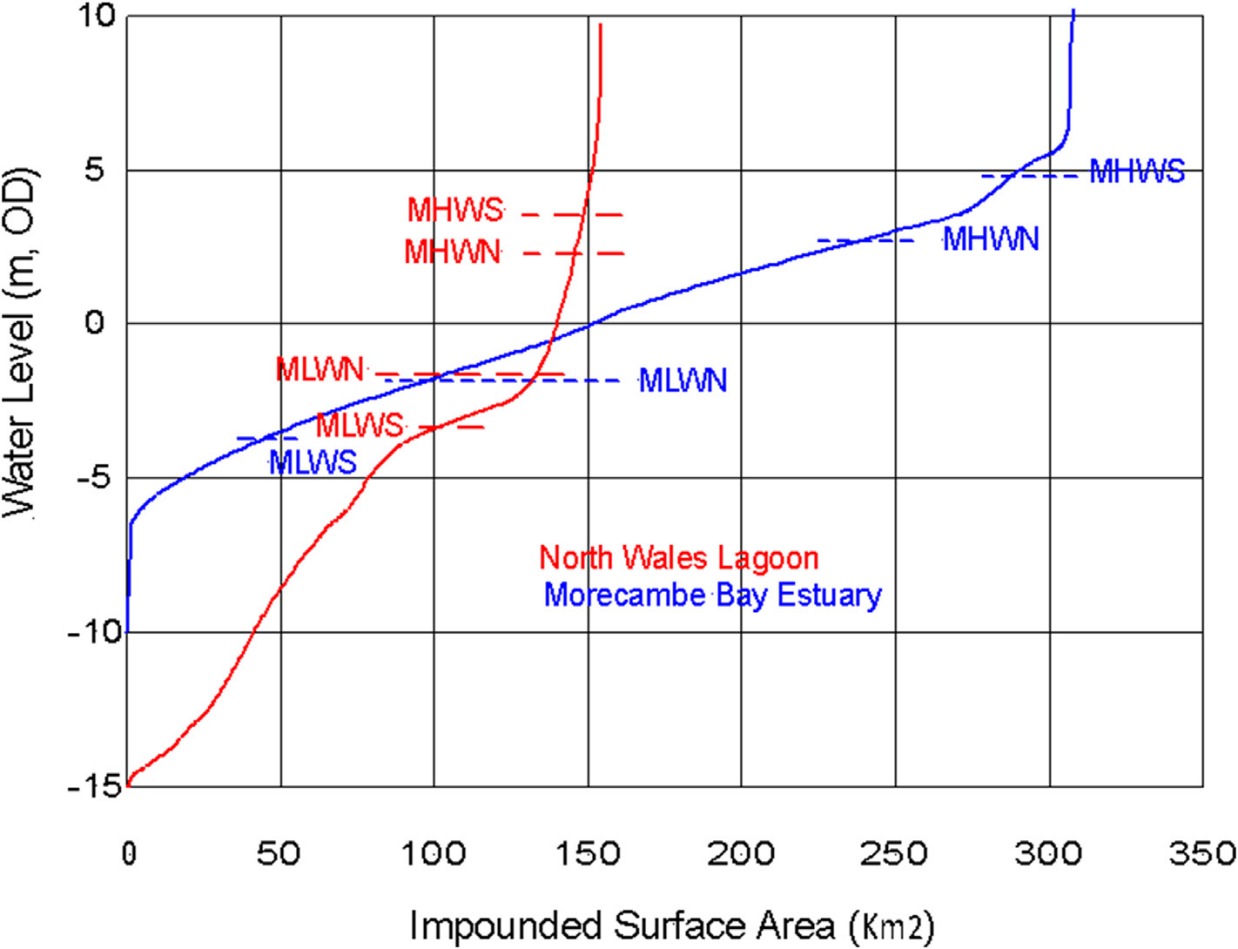
Impounded surface area by water level (MHWS – mean high water spring, etc.) for Morecambe Bay and North Wales proposals, after Baker [6] and Xue [7].

The reservoir definition is in the form of a function of impounded water surface area against water level. As no site-specific surveys are publicly available, both Baker [6] and Xue [7] formed 3-D digital terrain models from LIDAR data [20] supported by Admiralty charts [21]. A transposed version of their graphs showing water levels on the vertical axis is presented in Figure 4. The current mean spring tide levels have also been added for each site.

Figure 4 shows that the impounded surface water areas for spring tides in North Wales varies between 100 and 150 km^2^, whilst the corresponding range for Morecambe Bay is about four times larger (50–280 km^2^). The areas in Figure 4 between tide levels gives the volume of water moving with each tide, see Table 2.

**Table 2:** Tidal flows for Morecambe Bay and North Wales proposals.

	Volume of water flowing × 10^6^ m^3^	
	North Wales	Morecambe Bay
Mean Spring tides	937	1,440
Mean Neap tides	540	721

It should be noted that the wetted area functions are significantly different between the two example sites. The maximum area enclosed by the NW lagoon is about half that of Morecambe Bay. However, the average wetted area is approximately the same, i.e., a large proportion of Morecambe Bay drains to expose mudflats and sand banks at low tide while for North Wales exposes only 33% of its area at low tide, as mostly sandy beaches.

## 4 Electrical generation

The 0-D model is ideal for initial assessment of schemes to allow comparison between sites. It is recognised that 0-D models can overestimate the total generation compared with more detailed 2-D or 3-D models [22]. However, Angeloudis found that the difference for his Clwyd Lagoon was < 10% [23]. Detailed surveys and 3-D modelling are needed to identify the optimum locations of turbines and sluices for the best environmental outcomes, e.g., managing sediment flows, avoiding stagnant water, maintaining oxygen levels and maximising habitat creation/stability.

Commonly, pumping is reported to increase the nett annual energy output by approximately 10% [24]. Pumping takes place at low heads after slack tide to increase the head available during the next generation sequence. However, it needs to be clear what is meant by pumping, as there are several different management schemes. For 2-way generation without pumping, the range of water levels inside the impoundment is less than the original tide range over the same period as the equalisation of water levels occurs after high or low tide. One pumping scenario is to pump to the natural tide level for each cycle to preserve the intertidal area. This is the method used in the following analysis.

### 4.1 Number of turbines and AEP

**Figure 5:**
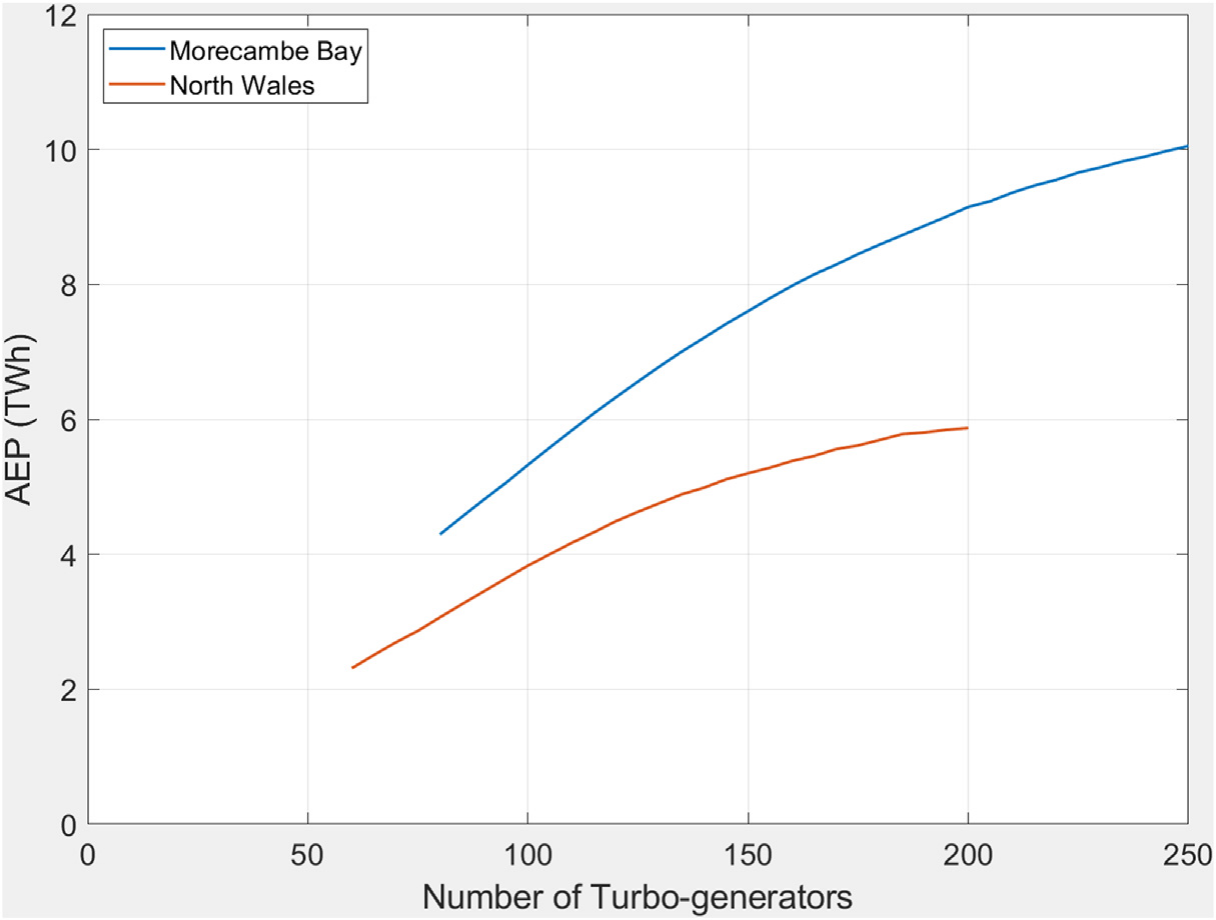
Modelled Annual Energy Production for Morecambe Bay and North Wales for a range of 8m diameter turbines.

The 0-D model has been used to estimate the annual energy generation for varying numbers of 8m diameter turbines (see Figure 5). The Morecambe Bay curve is based on 30-MW generators, and that for North Wales on 20-MW. The resulting curves are asymptotic showing a gradual flattening off as the number of turbines increase; the theoretical maximum for a cycle requires the reservoir to be exhausted instantly over the full tidal range, achievable only with an infinite number of turbines. There is broad scope to choose how much electricity is generated determined by the number of turbines installed. Based on purely economic terms, the best solution might be determined by the balance of value of electricity generated against the costs of construction and operation; the major factors include the price of the turbines and the estimated returns from the sale of electricity.

**Figure 6:**
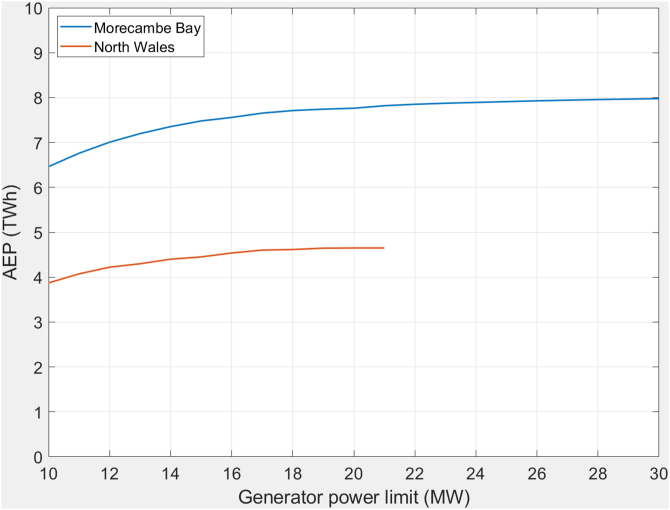
Predicted annual generation (TWh) plotted against generator rating (MW).

The sluice capacity and generator sizing are additional factors to consider at the early stages of the design that also strongly effect the economics. The generator accounts for a significant part of the cost of turbogenerators so it is desirable not to over specify the size of generator required. Figure 6 shows the annual energy produced from both schemes for 120 turbines in North Wales and 160 in Morecambe Bay; plotted against generator maximum power rating. The numbers of turbines correspond to equivalent resource utilisation (percentage of theoretical maximum available energy). For North Wales there is little increase in AEP observed above 18-MW, and a rating below this may be more economic. For Morecambe Bay there is only a small increase over 22-MW. The tidal range limits the operating head and speed of the turbines. Thus, there is a practical limit on the generator rating for each proposed scheme. Reducing the generator rating specified will reduce capital cost of the generators and electrical equipment significantly.

**Figure 7:**
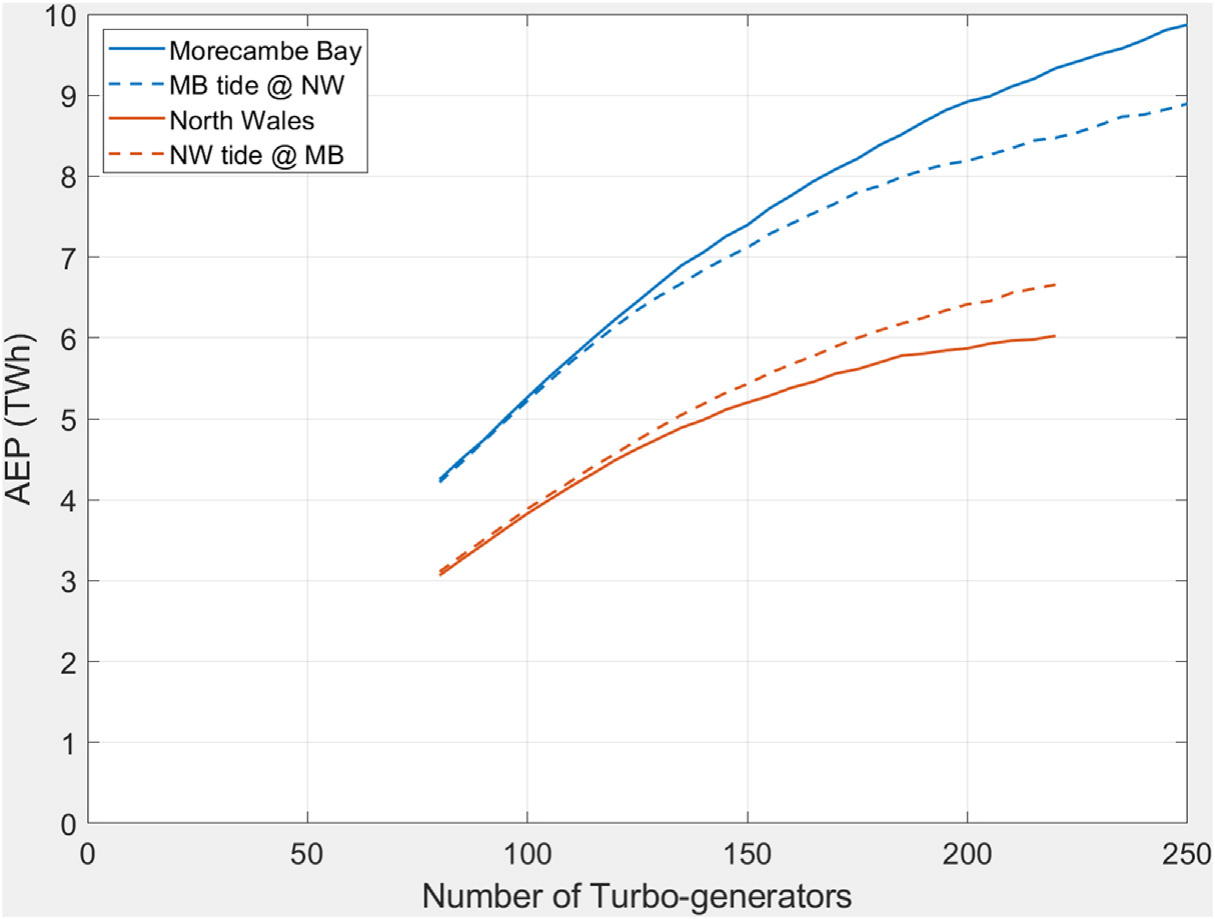
Predicted annual generation for NW and MB schemes each modelled using both tide sets. Tidal range is a dominant factor for AEP.

It is no surprise that Morecambe Bay can generate more energy than North Wales since it has a greater tidal range (Table 1) and greater flow of water between tides (Table 2). To remove the effect of different tidal range and volume the Morecambe Bay tidal range was applied to the North Wales coastal lagoon bathymetry and vice versa; the results are shown in Figure 7. The solid lines are for schemes modelled with their respective tide, the dashed lines are the cases modelled with the opposing tide, and the colour represent the tide – blue for Morecambe Bay and brown for North Wales. The tidal range is the dominant factor, with the NW tide providing approximately 75% of the energy compared to the MB tide. The difference due to the reservoir is secondary, with NW approximately 10% lower than MB. This can be seen in the separation between the two at large numbers of turbines; limited numbers of turbines prevent the reservoir from being fully exploited and preferentially depress the output from the larger reservoir, hence the curves converge for fewer turbines.

**Figure 8:**
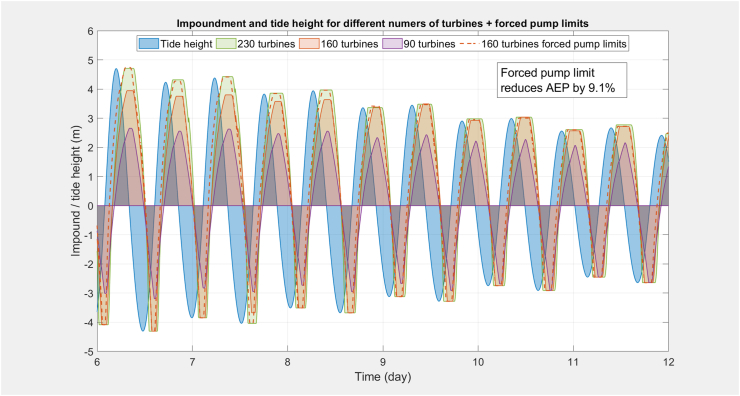
Modelled tide, impoundment water levels for 6-days at Morecambe Bay, with 30-MW generators with various numbers of turbines and a sluice ratio = 1. With enough turbines, it is possible to maximise AEP and achieve tide limit matching. With an intermediate number of turbines, modification to the operation is required to achieve tide limit matching, which incurs a penalty in lost AEP. With too few turbines, it is impossible to achieve tide matching.

Beyond the economics, there may be additional constraints on the number of required turbines. Figure 8 shows 0-D model output of sea and impoundment levels for a typical 6-day spring/neap cycle for Morecambe Bay. The blue curve shows the sea level, the remaining curves are the impoundment water level for different numbers of turbines. With enough turbines, it is possible to maximise AEP and achieve tide limit matching. With an intermediate number of turbines, modification to the ebb generating cycle start and stopping heads are required to ensure the equilibrium point (tide = barrage height) is close enough to high tide to enable the pump to achieve the tide limits before the pump maximum head is reached. There is a penalty in lost AEP of 9.1% in the example of 160 turbines shown here. With a small number of turbines, the discharge rate during generation is insufficient to achieve equilibrium close enough to high tide and it is impossible to achieve tide matching. This demonstrates that loss of inter-tidal area for 2-way generation can be avoided, provided there are enough machines installed.

### 4.2 Reservoir topography – impact on the ebb and flood cycles

The reservoir topography effects the characteristics of the ebb and flood power generation cycles. For a perfect lagoon, with vertical sides and where the impounded water surface area remains constant regardless of the water level, the ebb and flood generating cycles will be equal for the same tidal cycle. Typically, an estuarine reservoir will have a larger impound at high-water compared to its low water than a lagoon with a similar shoreline topography as it has approximately double the length of coastline. Consequently, the ebb and flood generating cycles will differ. The intertidal area (i.e., the area that is inundated and exposed during the cycle) determines how quickly the impounded water level changes for a given discharge rate through the turbines. At the start of the ebb cycle where the area is larger, the height drops relatively slowly compared with the start of the flood cycle when the area is much smaller, and the height rises quickly. The relative change in impoundment and tide height determines how the head changes; in the ebb cycle the initial head change is smaller than for the flood cycle, allowing the ebb generating cycle to operate over a higher average head and generate more energy.

**Figure 9:**
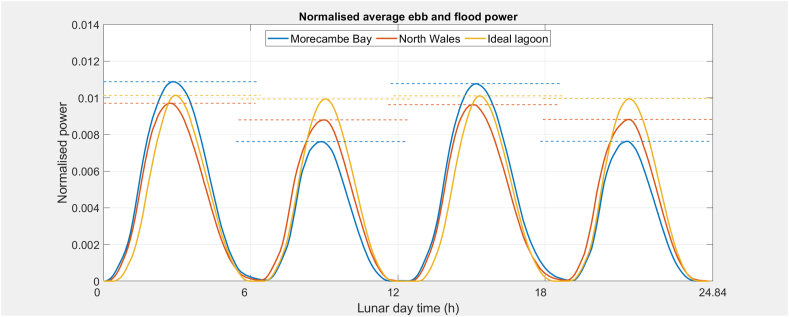
Normalised mean lunar day power generation for a full year of operation (354 lunar days) showing a sequence of ebb-flood-ebb-flood generating cycles. Morecambe Bay, with a greater variation in impound surface area vs height shows a larger variation between ebb and flood cycles compared to North Wales. The idealised lagoon has almost equal ebb and flood cycles; a slight difference is expected due to the tide-to-tide fluctuations in amplitude caused by the higher frequency tidal harmonic constituents.

Figure 4 shows the tidal height vs impounded area functions for Morecambe Bay and North Wales that are used to describe the respective reservoirs in the 0-D modelling. With a much larger variation in exposed area of the two, it is expected that Morecambe Bay will experience a greater difference between ebb and flood tides. Figure 9 is a graph of the normalised mean lunar day power generation for a full year of operation (354 lunar days) for a sequence of ebb-flood-ebb-flood generating cycles for Morecambe Bay, North Wales and an ideal lagoon (constant area vs height function with equivalent AEP). The same tidal sequence has been used in all cases and the number of turbines has been determined to give the same overall reservoir resource utilisation. Morecambe Bay, with a greater variation in impound surface area vs height shows a larger variation between ebb and flood cycles compared to North Wales. The idealised lagoon has almost equal ebb and flood cycles; a slight difference is expected due to the tide-to-tide fluctuations in amplitude caused by the higher frequency tidal harmonic constituents.

**Figure 10:**
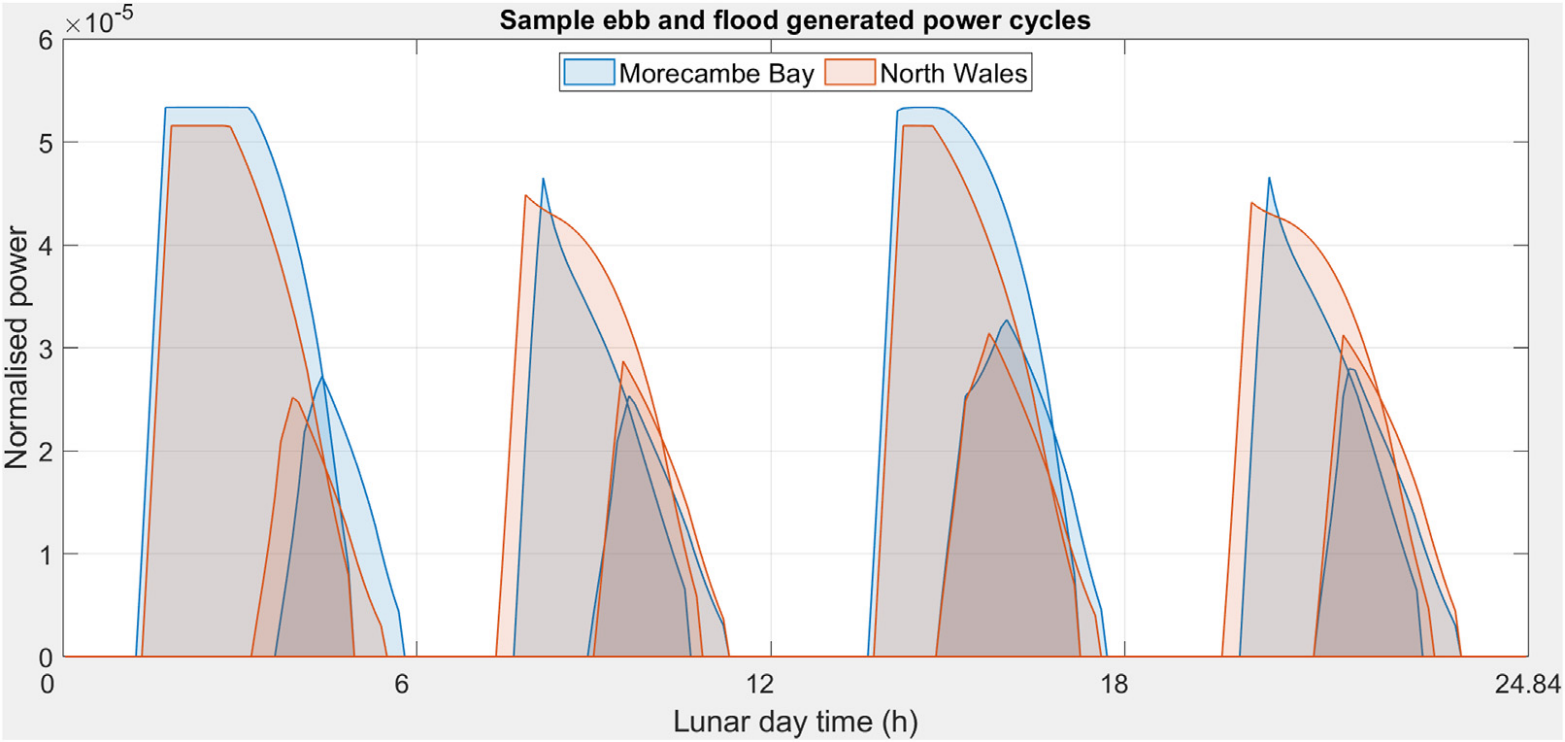
Sample individual lunar day generating cycles showing a sequence of ebb-flood-ebb-flood generating cycles for spring, and neap tides (higher tide height corresponds to higher power generation): the flood cycles show the abrupt decline in power after the initial peak from the rapidly declining head – less so for NW than MB; the ebb cycles show an increase in power after the initial peak indicating the head is still increasing due to a more rapidly changing tide height than the impound. Note tides in sets of 4, the first 2 are ebb and second 2 are flood.

Figure 10 shows sample individual lunar day generating cycles for a sequence of ebb-flood-ebb-flood spring and neap tides. The flood cycles show an abrupt decline in power after the initial peak, a result of the rapidly declining head, and is more pronounced for MB than NW. The ebb cycles show an increase in power after the initial peak, indicating the head is still increasing due to a more rapidly changing tide height than the impound height. Significantly more energy is generated during spring tides than neap tides, from both higher power and increased generating cycle duration. The flat top to the spring cycle peaks reflects the maximum power limit of the turbines has been reached.

## 5 Ecology and the environment

The primary objective of most developers of tidal range schemes is to maximise the generation of clean, green energy by exploiting an inexhaustible and freely available source. However, there are multiple drivers for development and nowadays some carry as much weight, if not more, than power generation. Environmentalists have considerable concerns over conservation and habitat protection; governments guarantee to protect these through international agreements. Social scientists prioritise flood protection, poverty reduction and economic regeneration through the creation of jobs, whilst recreational opportunities and improved transport tends to be of more importance to the local economy. Economists commonly try to balance and express all the costs and benefits in terms of finance but there is currently no specific funding for these other benefits. The situation is not stable, flood prevention and conservation of the inter-tidal zone are becoming more important with sea-level rise.

It may seem counterintuitive that building an impoundment, with inevitable disruption to the ecology, will be beneficial to the environment. However, the potential damage through sea-level rise, if nothing is done, will be far greater than the short-term impacts of development. The balance of ecological damage and gains can be viewed as costs and benefits needing comprehensive detailed examination. The risks are difficult to evaluate in comparison to the magnitude of threats forecast from climate change models. One climate change consequence, sea-level rise, is widely accepted and already being observed [9]. The inter-tidal zone will be pushed further inshore, where it will meet man-made resistance, in the form of flood prevention embankments, preventing its natural migration. Mud flats will remain submerged longer; the area of salt marsh will shrink as it remains inundated longer. Near shore habitats will be protected from sea level rise by man-made defences forcing a reduction in extent of intertidal systems even putting them at risk of becoming lost altogether. Saltmarsh (Figure 2) is now recognised as an important carbon sink and should be protected where possible [25].

For the ecology, it is important to know which habitats and species are present, their current extent, condition and dynamics. Unfortunately, this is under-recorded. Fish and birds are often reported more effectively, but their supporting community species are usually ignored. Every proposed scheme will need comprehensive surveys of the site-specific ecology during the feasibility stage so that plans can be made to minimise disruption during construction and maximise the benefits during operation.

As shown in Table 3, the estuarine area usually includes highly diverse habitats recognised as of great significance to wildlife, consequently they are the most designated and protected areas in the UK. The presence of an impoundment can change the nature of the inter-tidal zone within it, but it can also be managed as an environmental protection scheme. Importantly, a barrage can limit the height of the high tides to alleviate tidal flooding and mitigate riverine flooding, maintaining the current tidal range, thus preserving conditions for existing habitats. From Figure 4, a 1m rise in MLWS would lose 27-km^2^ (approximately 10%) of Morecambe Bay's intertidal area. A 2m rise would lose 56-km^2^ of “protected” area. Through international agreements the UK Government (e.g., the RAMSAR agreement) has accepted responsibility to protect its designated areas; barrages are the only affordable form of protection that can be delivered in a reasonable timeframe, but only if we start now.

**Table 3:** Percentages of different designated areas covering Morecambe Bay and North Wales lagoon.

		North Wales		Morecambe Bay	
		Barrage	+5km buffer	Barrage	+5km buffer
Area (km^2^)		159.8	578.4	306.5	928.4
Costal length (km)		37.26	–	153.58	–
Bund length (km)		33.65	–	18.73	–
Flood prevention area (km^2^)		33.02	–	67.83	–
Designated protection areas (%)	Ramsar	0.0%	1.2%	83.6%	32.4%
	SSSI	0.4%	4.3%	83.7%	36.1%
	SAC	14.2%	11.7%	98.7%	45.8%
	SPA	94.5%	60.1%	86.6%	35.3%
	AONB	0.0%	2.3%	10.7%	8.4%
	NNR	0.0%	0.0%	0.0%	1.2%
	RSPB	0.0%	0.0%	8.3%	3.1%
	Salt marsh	0.0%	0.0%	4.8%	2.4%
	No designation	5.2%	34.7%	0.8%	46.3%

There is 14.7 km^2^ of designated saltmarsh around Morecambe Bay. If this were all to be lost due to sea-level rise it would not only represent a conservation loss in capacity to capture carbon and sequester (8.0 tCO_2_/ha/yr ≥ 1.176 Mt/yr); potentially there will be a release of the stored carbon (Salt marsh: 917 Mg CO_2_e/ha ≥ 134.8 Mt) [25].

## 6 Discussion and conclusions

The question of the difference between coastal tidal lagoons and estuarine barrages as a method of power generation has several levels of importance. As government is seeking to guarantee sustainable power from sources within its control, it must consider tidal and wave energy options. Where public funds are provided, decisions must be supported by transparent and comparable information and the importance of multi-functionality considered. For developers, the rate of financial return compared to the outlay, the payback period, size of financial support, additional sources of income and security are all important. Whilst the public and politicians demand green credentials and social benefits.

As mentioned in the introduction there are several preconceptions that suggest the two forms of scheme are different. In terms of landscape morphology, the difference between the two types of sites can be characterised by describing barrages on estuaries as closing an open ‘V’ whilst bunds on coastal lagoons form the curved section of a ‘D’; real schemes will not match this classification description perfectly. The volume of the impoundment is dependent not only on the two-dimensional shape but also the bathymetry and intertidal area.

Estuaries are defined as the mouth of large rivers as they enter the sea. Even with Britain's large rivers, the volume of water entering the impoundment from the land side per cycle is negligible compared to the tidal volume. Coastlines without estuaries will have direct runoff from the land along with smaller rivers and streams, making the difference between the schemes even smaller.

As demonstrator sites the two selected each have their own idiosyncrasies, with Morecambe Bay (MB) being more ‘C’ than ‘V’ shaped and North Wales (NW) being more ovoid than ‘D’. The two schemes, however, differ greatly in terms of area, coastal length, bund length and potential flood prevention area, see Table 3. The information is important when comparing the sites.

To produce a more representative analysis both sites were analysed with their own tidal regimes and that of the other site. The analysis shows that estuaries will produce greater output per annum, due in large part to the funnelling of water forming a higher head. There is an interesting interaction with the ebb and flood tides with estuaries showing a greater range (Figure 10), higher on the ebb but lower on the flood. The spring tides are marginally higher for estuaries, but not significantly. Without effective power storage, the timing of generation in relation to demand is important. The timing of the spring tides for both sites is currently well matched to demand, suggesting that it is a small benefit for an estuary, but the timing of demand may vary, especially with the increase in electric vehicles.

Coastal lagoons have become more popular of late since estuarine barrages were considered to have a negative impact on ecology and the environment. In contrast, tidal lagoons were seen to occupy ecologically less valuable space and therefore have less resistance to development. In reality, the ecological and environmental value of non-estuarine coastline probably has similar range of values but is so far largely un-assessed. Available knowledge of the ecology and dynamics of the white ribbon of intertidal and near-shore features is patchy and needs improvement. Neither estuarine barrages nor coastal lagoons can be described as of greater ecological value simply based on their form and level of designation.

Now that sea-level rise is a fact to be dealt with, the barrages may offer a way of protecting these important areas. Current designation of sites is highest in estuaries and some designations (e.g., RAMSAR, SAC and SPA) are covered by international agreements obliging the government to protect of create similar quality of sites elsewhere. The simulations run here were constrained to maintain the existing range offering protection with a nett benefit to ecology and the environment due to the maintenance of intertidal area despite rising sea levels. Further research is urgently needed so that engineers, ecologists, and other sciences can work together to find an equitable solution.

In terms of coastal flooding, both systems are vulnerable and will benefit from protection. Estuaries may be at greater risk from and contribute to riverine flooding with high tides backing up rivers in spate. The presence of a barrage would allow the impoundment to be managed at a lower level by stopping the incoming tide and pumping the river water, using the turbines as pumps.

Estuaries may benefit from greater opportunities for funding and support for other functions. With transport, lagoons lack the potential to link two coastal areas, but as a consequence the bund would be narrower employing less material per unit length. Both schemes will benefit from jobs and coastal towns are commonly economically poor. Currently the government advice [2] is to not consider coastal lagoons for hybrid status.

Other methods of generation or energy storage should be investigated in combination with tidal range. The barrage could form a secure foundation for large wind turbines, but it may not be safe to site them close to a road along the estuary barrage. For the coastal lagoon the wind turbines would only be 5km offshore and likely to be visually intrusive. Siting wave energy devices along the barrage should be investigated to see how much energy could be obtained; they may also dampen the wave forces on the barrage. Any surplus energy could be converted to hydrogen for long term storage or compressed air for short term storage and providing some measure of continuous generation. The compressed hydrogen or air can be stored in adapted caissons. Tidal steam units could be sited near the outfalls to capture additional energy and dissipate the high velocity water streams.

Estuarine barrages and coastal lagoons sit on a continuum of costs and benefits and need to be assessed openly and robustly to make comparisons.

In summary

• Estuarine barrages and tidal lagoons are similar and complimentary. The biggest factor for power generation is the tidal range.
• The benefits of a bund can include protecting the environment, population, transport, housing, recreation, conservation, and business.
• Assessment of schemes needs to focus on more than simply power generated.

## Declarations

### Author contribution statement

David Vandercruyssen: Conceived and designed the experiments; Performed the experiments; Analyzed and interpreted the data; Wrote the paper.

Simon Baker: Contributed reagents, materials, analysis tools or data.

David Howard; George Aggidis: Conceived and designed the experiments; Wrote the paper.

### Funding statement

This research did not receive any specific grant from funding agencies in the public, commercial, or not-for-profit sectors.

### Data availability statement

Data included in article/supp. material/referenced in article.

### Declaration of interest’s statement

The authors declare the following conflict of interests: David Vandercruyssen; First author is also a director of North Wales Tidal Energy, as stated in acknowledgements.

### Additional information

No additional information is available for this paper.
